# Cancer-derived exosomes from HER2-positive cancer cells carry trastuzumab-emtansine into cancer cells leading to growth inhibition and caspase activation

**DOI:** 10.1186/s12885-018-4418-2

**Published:** 2018-05-02

**Authors:** Mark Barok, Maija Puhka, Gyorgy Vereb, Janos Szollosi, Jorma Isola, Heikki Joensuu

**Affiliations:** 1 Laboratory of Molecular Oncology, University of Helsinki, Biomedicum Helsinki, Haartmaninkatu 8, FIN-00290 Helsinki, Finland; 2 Institute for Molecular Medicine FIMM and EV Core, University of Helsinki, Helsinki, Finland; 3 Department of Biophysics and Cell Biology, Faculty of Medicine, University of Debrecen, Debrecen, 4032 Hungary; 4 MTA-DE Cell Biology and Signaling Research Group, Faculty of Medicine, University of Debrecen, Debrecen, 4032 Hungary; 5 Laboratory of Cancer Biology, Medical Faculty, University of Tampere, Biokatu 6, 33014 Tampere, Finland; 6 Department of Oncology, Helsinki University Hospital and University of Helsinki, Haartmaninkatu 4, FIN-00029 Helsinki, Finland

**Keywords:** Breast cancer, Trastuzumab-emtansine, T-DM1, HER2, Exosome

## Abstract

**Background:**

Trastuzumab emtansine (T-DM1) is an antibody-drug conjugate that carries a cytotoxic drug (DM1) to HER2-positive cancer. The target of T-DM1 (HER2) is present also on cancer-derived exosomes. We hypothesized that exosome-bound T-DM1 may contribute to the activity of T-DM1.

**Methods:**

Exosomes were isolated from the cell culture medium of HER2-positive SKBR-3 and EFM-192A breast cancer cells, HER2-positive SNU-216 gastric cancer cells, and HER2-negative MCF-7 breast cancer cells by serial centrifugations including two ultracentrifugations, and treated with T-DM1. T-DM1 not bound to exosomes was removed using HER2-coated magnetic beads. Exosome samples were analyzed by electron microscopy, flow cytometry and Western blotting. Binding of T-DM1-containing exosomes to cancer cells and T-DM1 internalization were investigated with confocal microscopy. Effects of T-DM1-containg exosomes on cancer cells were investigated with the AlamarBlue cell proliferation assay and the Caspase-Glo 3/7 caspase activation assay.

**Results:**

T-DM1 binds to exosomes derived from HER2-positive cancer cells, but not to exosomes derived from HER2-negative MCF-7 cells. HER2-positive SKBR-3 cells accumulated T-DM1 after being treated with T-DM1-containg exosomes, and treatment of SKBR-3 and EFM-192A cells with T-DM1-containing exosomes resulted in growth inhibition and activation of caspases 3 and/or 7.

**Conclusion:**

T-DM1 binds to exosomes derived from HER2-positive cancer cells, and T-DM1 may be carried to other cancer cells via exosomes leading to reduced viability of the recipient cells. The results suggest a new mechanism of action for T-DM1, mediated by exosomes derived from HER2-positive cancer.

**Electronic supplementary material:**

The online version of this article (10.1186/s12885-018-4418-2) contains supplementary material, which is available to authorized users.

## Background

Trastuzumab emtansine (T-DM1, Kadcyla^®^), an antibody-drug conjugate (ADC), was designed to deliver the cytotoxic drug DM1 (a derivative of maytansine) specifically to human epidermal growth factor receptor-2 (HER2)-positive cancer cells. In T-DM1, DM1 molecules are conjugated to the humanized monoclonal HER2-binding antibody trastuzumab via a non-reducible thioether linker SMCC (*N*-succinimidyl-4-(N-maleimidomethyl) cyclohexane-1-carboxylate; MCC after conjugation). One T-DM1 molecule carries an average of 3.5 DM1 molecules per one molecule of trastuzumab [1]. Binding of T-DM1 to HER2 receptors evokes receptor-mediated endocytosis of the T-DM1-HER2 complex [2, 3], which is followed by the proteolytic degradation of the antibody part of T-DM1 in the lysosomes resulting in a release of lysine-MCC-DM1 into the cytosol [1]. Lysine-MCC-DM1 is a potent inhibitor of the microtubule assembly leading to cancer cell death [4, 5]. T-DM1 had substantial anti-cancer efficacy in preclinical models of HER2-positive breast cancer [1, 6], and after demonstration of its efficacy and safety in clinical trials [7, 8], the U.S. Food and Drug Administration (FDA) approved T-DM1 in 2013 as monotherapy for the treatment of patients with HER2-positive advanced breast cancer who had previously received trastuzumab and a taxane.

Exosomes are small (30 to 200 nm) extracellular vesicles covered by a lipid bilayer membrane. They are generated by exocytosis of multivesicular bodies [9]. Exosomes may act as cell waste bags, but they may also have a function in cell-cell communication as their contents (e.g. proteins, lipids, various RNA species and DNA) can be carried both to the neighboring cells and distant cells [10–12]. All types of cells, including cancer cells, secrete exosomes. Exosomes secreted by cancer cells are called cancer-derived exosomes [9]. Cancer-derived exosomes have been implicated in the modulation of cancer growth and metastasis, tumor angiogenesis, and anti-cancer immunity [13, 14].

HER2, the target protein of T-DM1, may be present on cancer-derived exosomes [15, 16]. We hypothesized that the anti-HER2 ADC, T-DM1, might also bind to cancer-derived exosomes. Since exosomes can carry their contents into other cells [13], HER2-positive cancer-derived exosomes might carry the cytotoxic drug DM1 to distant cells, with potential drug safety and efficacy consequences. Despite the efficacy of T-DM1, most patients treated with T-DM1 progress, and some HER2-positive breast cancers are primarily resistant to T-DM1 [7, 8]. The mechanisms of resistance are incompletely understood [4], and treatment options for patients with T-DM1-resistant tumors are limited. Therefore, revealing new mechanisms of action of T-DM1 might have clinical relevance. In this study we show that T-DM1 binds to exosomes derived from HER2-positive breast and gastric cancer cells and that HER2-positive breast cancer cells take up T-DM1 carried on cancer-derived exosomes. Treatment of HER2-positive breast cancer cells with T-DM1-exosomes resulted in caspase activation and death of cancer cells. To our knowledge, this is the first study to report on the biological effects of exosome-bound anti-HER2 agents on cancer cell apoptosis.

## Methods

### Cell lines and antibodies

The human breast cancer cell line EFM-192A was obtained from the German Resource Center for Biological Material (DSMZ, Braunschweig, Germany), the MCF-7 and SKBR-3 human breast cancer cell lines from the American Type Culture Collection (ATCC, Manassas, VA), and the SNU-216 human gastric cancer cell line from the Korean Cell Line Bank (Seoul, Korea). The MCF-7 cell line is HER2-negative, whereas SKBR-3, SNU-216, and EFM-192A overexpress HER2. The SKBR-3 and EFM-192A cell lines are sensitive to T-DM1, whereas the SNU-216 and MCF-7 cells are insensitive [6, 17] (Table 1). The cell lines were cultured according to recommended specifications. Trastuzumab-DM1 was purchased from Roche Ltd. (Basel, Switzerland). Covalent binding of Alexa Fluor 488 (A488) (Invitrogen/Thermo Fisher Scientific, Carlsbad, United States) to T-DM1 was carried out according to the manufacturer’s instructions. The dye to protein labeling ratio was approximately 1:1.5.

**Table 1:** The cell lines investigated

Cell line	Origin	HER2 status	Reported sensitivity to T-DM1
SKBR-3	Human breast cancer	Positive	Sensitive (IC_50_ 0.005 μg/mL) [1, 6]
EFM-192A	Human breast cancer	Positive	Sensitive (IC_50_ 0.426 μg/mL) [6]
SNU-216	Human gastric cancer	Positive	Insensitive (IC_50_ > 10 μg/mL) [17]
MCF-7	Human breast cancer	Negative	Insensitive (IC_50_ > 10 μg/mL) [6]

IC_50_, half-maximal inhibitory concentration

### Preparation of exosome-depleted cell culture medium

As fetal bovine serum (FBS) contains substantial amounts of bovine exosomes [18], exosome-depleted medium was prepared as described previously [19]. In brief, cell culture medium supplemented with all nutrients and 20% FBS was centrifuged overnight at 100,000×g at 4 °C. The ultracentrifugation was done in polycarbonate tubes (26.3 mL, Beckman Coulter, Brea, USA) with a 50.2 Ti fixed-angle rotor (Beckman Coulter) in an Optima XL-80 K Ultracentrifuge (Beckman Coulter). The supernatant, which equals the exosome-depleted medium, was filtered through a 0.22-μm filter (Merck Millipore, Billerica, USA). The exosome-depleted medium was diluted with a medium supplemented with all the nutrients and antibiotics, but no FBS, in order to reach about 10% FBS concentration. The exosome-depleted medium was stored at 4 °C until use.

### Isolation of exosomes from the cell culture media and fetal bovine serum (Type A exosomes)

**Fig. 1:**
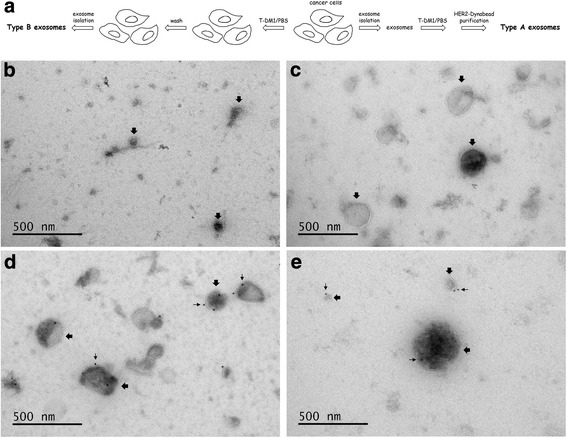
Isolation and treatment of Type A and Type B exosomes (**a**). T-DM1 binds to Type A exosomes prepared from the cell culture medium of HER2-positive breast and gastric cancer cells. Immuno-electron microscopy images showing MCF-7 exosomes treated with T-DM1 (**b**), SKBR-3 exosomes treated with PBS (**c**), SKBR-3 exosomes treated with T-DM1 (**d**), and SNU-216 exosomes treated with T-DM1 (**e**). T-DM1 is present on exosomes derived from the HER2-positive cell lines (SKBR-3, SNU-216) and treated with T-DM1 (**d**, **e**), but not in the controls (**b**, **c**). Large arrows point at exosomes, small arrows at immuno-gold labelled T-DM1

Exosomes were first isolated from the cell culture media of SKBR-3, SNU-216, and MCF-7 cell lines. The cells were plated into cell culture flasks with a 175 cm^2^ surface area, washed with phosphate-buffered saline (PBS) three times the next day, and exosome-depleted cell culture medium was added into the bottles. The medium was harvested when the cell confluency reached about 90%. The harvested medium was purified by serial centrifugations (1000 × g for 15 min, 2000 × g for 35 min, and 10,000 × g for 35 min). The exosomes were isolated from the remaining supernatant by ultracentrifugation (100,000 × g for 150 min), then suspended in PBS followed by one additional ultracentrifugation (100,000 × g for 150 min). The pellet was suspended in 300 μL PBS, and then stored in Protein LoBind tubes (Eppendorf, Hamburg, Germany) at − 80 °C. All centrifugations were done at 4 °C. The ultracentrifugations were done in polycarbonate tubes (26.3 mL, Beckman Coulter, Brea, USA) with a 50.2 Ti fixed-angle rotor (Beckman Coulter) in an Optima XL-80 K Ultracentrifuge (Beckman Coulter) [19]. Since HER2 is not present on the FBS exosomes [20], we prepared exosomes also from cell culture media containing FBS by serial centrifugations as described above, and used them as a negative control. Hence forward, exosomes produced from cancer cells that have not been exposed to T-DM1 and those present in FBS are called “Type A” exosomes (Fig. 1a).

### Isolation of newly formed exosomes from the SKBR-3 cell line treated with T-DM1 (Type B exosomes)

To obtain exosomes for testing a hypothesis whether SKBR-3 cells treated with T-DM1 produce T-DM1-containing exosomes, we first plated SKBR-3 cells into culture flasks with a 175 cm^2^ surface area. When confluency reached about 75%, the cells were washed with PBS followed by incubation with either PBS or T-DM1 (25 μg/mL) or Alexa Fluor 488 (A488)-T-DM1 (25 μg/mL) at 4 °C for 30 min. The cells were then washed with PBS three times and incubated in an exosome-depleted cell culture medium overnight. The medium was harvested and the newly formed exosomes were purified by serial centrifugations as described above. We call these newly formed exosomes, secreted by cancer cells exposed to T-DM1, as “Type B” exosomes in the present study (Fig. 1a), to distinguish them from the exosomes secreted by cells that have not been exposed to T-DM1, and from the exosomes present in FBS (Type A exosomes).

### Purification of Type A exosomes with HER2-coated magnetic beads

Type A exosomes were incubated either with PBS or T-DM1 (25 μg/mL) at 4 °C for 30 min. T-DM1 that was not bound to exosomes was removed using HER2-coated magnetic beads. Human HER2 Protein (Sino Biological, Beijing, China) was coupled to magnetic Dynabeads using the Dynabeads^®^ Antibody Coupling Kit (Thermo Fisher Scientific, Waltham, USA) according to the manufacturer’s instructions. 100 μL of the exosome-containing sample mixed with T-DM1 was incubated with HER2-coated Dynabeads (286 μL; contains 14.3 μg HER2 and 2.95 mg Dynabeads). The appropriate amount of HER2-magnetic beads was determined using T-DM1-spiked PBS samples (data not shown). During the incubation, T-DM1 that was not bound to exosomes was captured by the HER2-coated Dynabeads. A magnetic field was next applied, and the supernatant was removed. The supernatant contained the exosomes, whereas the non-exosome bound T-DM1 remained on the HER2-Dynabeads.

### Flow cytometry

After incubation with T-DM1 and extraction of unbound T-DM1 with HER2-coated Dynabeads, the T-DM1 content of Type A exosomes was measured using flow cytometry [19, 21]. A volume of 38 μL each sample was incubated with 3 μL aldehyde/sulfate latex beads (4 μm; Thermo Fisher Scientific, Waltham, USA) for 15 min at room temperature (RT). The aldehyde/sulfate latex beads are hydrophobic with a high density of aldehyde groups on the bead surface, which enables non-specific binding of exosomes and proteins to the latex beads. PBS was then added to the samples to a final volume of 1 mL, and the samples were incubated on a rotator wheel for 120 min at RT. The samples were then mixed with 110 μL of 1 M glycine and incubated for 30 min at RT, washed with PBS, and the beads were suspended and washed with PBS with 2% bovine serum albumin (BSA) three times. For staining, the beads were suspended in 100 μL PBS with 2% BSA containing 1 μL A488-goat anti-human-IgG (A488-GAHIG; Jackson ImmunoResearch, West Grove, USA) and incubated for 30 min at RT. Then samples were washed with PBS twice and suspended in 130 μL PBS. Exosomes from the cell culture medium of the SKBR-3 and SNU-216 cell lines and treated with PBS were used as the controls to the exosomes that had been treated with T-DM1. Type B exosomes isolated from the cell culture medium of SKBR-3 breast cancer cells treated with A488-T-DM1 or PBS were prepared similarly, except that 2 μg of purified exosomes were incubated with 3 μL aldehyde/sulfate latex beads and these samples were not labeled with A488-GAHIG. The fluorescence intensity (FI) of A488 was determined using an Accuri C6 Flow Cytometer (Accuri Cytometers, Inc., Ann Arbor, USA).

### Protein quantification

For the total protein content quantification the samples were mixed with RIPA buffer (Cell Signaling Technology, Danvers, USA), incubated for 2 min at RT, and then mixed with the Bradford Ultra reagent (Novexin, Cambridge, UK). Serially diluted bovine serum albumin samples were used as the standard. Absorbance was measured at 595 nm using a PHERAstar FS plate reader (BMG Labtech, Ortenberg, Germany). Sample protein concentration was determined from the standard curve and the absorbance values of the samples.

### Electron microscopy

The samples were prepared for electron microscopy and imaged as described elsewhere [22] using an immunostaining procedure. In brief, after paraformaldehyde fixation, the samples were blocked with 0.5% BSA in 0.1 M sodium phosphate buffer (pH 7.0) for 10 min RT, incubated with 12 nm colloidal gold conjugated goat-anti-human-IgG (gold-GAHIG) secondary antibody (Jackson ImmunoResearch, West Grove, USA) in the same sodium phosphate buffer for 30 min at RT, washed with the sodium phosphate buffer and deionized water, stained with uranyl acetate, and embedded in a methyl cellulose uranyl acetate mixture. The samples were viewed using either a Tecnai 12 (FEI Company, Eindhoven, The Netherlands) or a Jeol JEM-1400 (Jeol Ltd., Tokyo, Japan) transmission electron microscope operating at 80 kV.

### Western blotting

For Western blotting, 4 μL of Type A exosomes were mixed with RIPA buffer (Cell Signaling Technology, Danvers, MA, USA), then with Laemmli sample buffer containing β-mercaptoethanol (Bio-Rad, Oxford, UK), denatured at 95 °C for 5 min, loaded to Mini-PROTEANR TGX™ 10% gradient SDS-PAGE gels (Bio-Rad, Oxford, UK), and blotted on Immobilon-P membranes (Millipore, Bedford, MA, USA). Blocking (1 h, RT) and antibody incubations (the primary antibodies overnight at 4 °C; the secondary antibodies 1.5 h at RT) were performed in 5% non-fat powdered milk (Valio, Helsinki, Finland) in TBS and in TBS with 0.1% Tween-20, respectively. The anti-human CD63 antibody (EXOAB-CD63A-1; 1:1000) was purchased from System Biosciences (Palo Alto, USA), and the anti-bovine CD63 antibody (abx021473; 1:500) from Abbexa (Cambridge, UK). For detection, horseradish (HRP)-conjugated secondary antibodies (HRP-conjugated rabbit anti-mouse IgG, 1:20,000; HRP-conjugated goat anti-human IgG (for detection of T-DM1), 1:20,000, both from Jackson ImmunoResearch; and HRP-conjugated goat anti-rabbit IgG, 1:20,000, System Biosciences) and a 1:2 mixture of SuperSignal™ West Pico Chemiluminescent Substrate and SuperSignal™ West Femto Maximum Sensitivity Substrate (Thermo Fisher Scientific, Waltham, USA) were used.

### Confocal microscopy

SKBR-3 cells were plated on chambered cover glasses (Thermo Fisher Scientific, Waltham, USA) and treated with 2.5 μL of Type A exosomes, 2 μg of Type B exosomes, PBS, or 20 μg/mL of T-DM1. The exosome-treated cells were incubated overnight, whereas the PBS-treated and T-DM1-treated cells were incubated for 2 h at 37 °C in a CO_2_ incubator. The cells were washed three times with PBS, fixed with 3.5% formaldehyde-PBS, permeabilized (0.1% Triton X-100 in PBS), washed with 3% BSA-PBS twice, and incubated with A488-goat anti-human-IgG (1:100) diluted in 3% BSA-PBS for 20 min at RT. Finally, the cells were washed three times with PBS and stored in 1% formaldehyde-PBS. The nuclei were stained with DAPI (Thermo Fisher Scientific, Waltham, USA). A Zeiss LSM 780 confocal laser-scanning microscope (Carl Zeiss, Göttingen, Germany) was used to image the samples. Fluorescence images were taken as 1 μm optical sections using a Plan-Apochromat 63 × (numerical aperature, 1.4) oil immersion objective.

### In vitro assays of cell viability and caspase activation

The effects of T-DM1-exosomes on cell viability were assessed by the AlamarBlue method (Thermo Fisher Scientific, Waltham, USA). SKBR-3 and EFM-192A cells were trypsinised and plated in 96-well flat-bottomed tissue culture plates (8000 cells per well). After overnight culture, the medium was exchanged to a medium containing 2.5 μL of Type A exosomes or 11 μL of Type B exosomes. As a positive control, cells were treated with a medium containing 1 μg/mL T-DM1 [6]. The number of viable cells was assessed after a 96-h incubation followed by adding the AlamarBlue reagent (Thermo Fisher Scientific, Waltham, USA). Fluorescence was measured with excitation at 540 nm and emission at 590 nm using a PHERAstar FS plate reader (BMG Labtech, Germany). The fluorescence of the samples was normalized with fluorescence values of the cell culture media that did not contain cells. The results are presented as the proportion of viable cells, which was obtained by dividing the fluorescence of the test samples by the fluorescence of PBS-treated control samples.

To estimate the tendency of breast cancer cells to undergo apoptosis, caspase activation was assessed using the Caspase-Glo 3/7 method (Promega, Madison, USA). The cells were trypsinised and plated in 96-well flat-bottomed tissue culture plates, 8000 SKBR-3 cells or EFM-192A cells per well. After overnight culture, the medium was exchanged to a medium containing 2.5 μL of Type A exosomes. As a positive control, the cells were treated with a medium containing 1 μg/mL of T-DM1 [6]. After 96 h of incubation, 100 μL of the medium was transferred into white-walled 96-well plates, mixed with 100 μL Caspase-Glo 3/7 reagent, incubated for 30 min at RT, and the luminescence was recorded using a PHERAstar FS plate reader (BMG Labtech, Germany). The results are presented as luminescence units obtained after subtracting the luminescence value from a blank reaction (without T-DM1 treatment).

### Statistical analysis

The data are expressed as the mean ± standard error (SE). Groups were compared using the Student’s t test after verifying that the data passed the test for the normal distribution. *P* values ≤0.05 with 2-sided testing were considered significant.

## Results

### T-DM1 binds to Type A exosomes derived from HER2-positive breast and gastric cancer cells

Extracellular vesicles of 30 to 300 nm in diameter (called here as exosomes) were detected with transmission electron microscopy in the culture medium of MCF-7, SKBR-3, and SNU-216 cell lines, and in FBS (Fig. 1, Additional file 1: Figure S1). At immuno-electron microscopy, T-DM1 was present on the surface of Type A exosomes derived from the HER2-positive cell lines (SKBR-3, SNU-216) and treated with T-DM1, but not on any of the control Type A exosomes (SKBR-3 or SNU-216 exosomes treated with PBS, or MCF-7 or FBS exosomes treated with T-DM1).

**Fig. 2:**
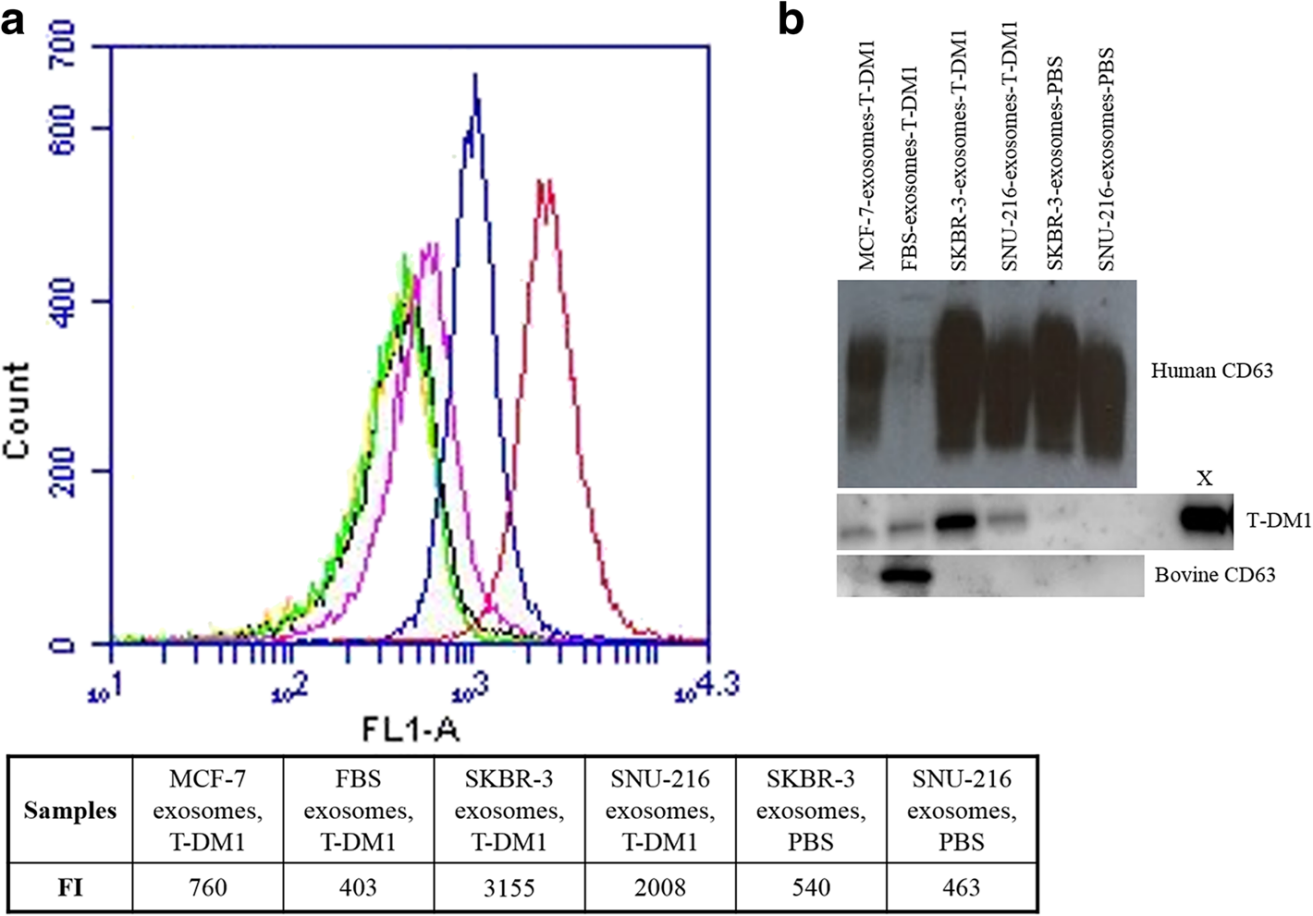
The T-DM1 and CD63 content of Type A exosomes. T-DM1-treated SKBR-3 and SNU-216 exosomes (red and blue, respectively) have a higher fluorescence intensity (FI) in flow cytometry indicating a higher T-DM1 content in these exosomes as compared with the control samples (T-DM1-treated MCF-7 exosomes, pink; T-DM1-treated FBS exosomes, green; PBS-treated SKBR-3 exosomes, orange; PBS-treated SNU-216 exosomes, black) (**a**). The human exosome marker protein CD63 is present in the Type A exosomes obtained from the culture media of the human cell lines, and the bovine CD63 exosome marker in FBS treated with T-DM1 in a Western blot analysis (**b**). T-DM1 content was high in SKBR-3 cell line-derived exosomes treated with T-DM1 (B). 55 ng of T-DM1 was used as a positive control (X)

In a flow cytometry analysis, where exosome-bound T-DM1 was detected by staining it with A488-goat anti-human IgG, high amounts of T-DM1 were found in Type A exosomes derived from the culture media of the HER2-positive cell lines (SKBR-3, SNU-216) and treated with T-DM1 compared to exosomes from the HER2-negative cell line MCF-7 or FBS treated with T-DM1, or to SKBR-3 or SNU-216 exosomes treated with PBS (Fig. 2a).

In a Western blot analysis using the human exosome marker CD63, Type A exosomes were detected in the culture media of all human cell lines tested. Bovine exosomes were detected in FBS with the bovine-specific antibody against exosome marker CD63 (Fig. 2b). A high T-DM1 content was found in SKBR-3 exosomes treated with T-DM1 and a lower content in SNU-216 exosomes treated with T-DM1. Small amounts of T-DM1 were detected also in two negative controls, in FBS exosomes and in MCF-7 exosomes treated with T-DM1, suggesting that some T-DM1 remained in these samples after the HER2-Dynabead purification.

### HER2-positive cells internalize T-DM1 after being treated with Type A T-DM1-exosomes

**Fig. 3:**
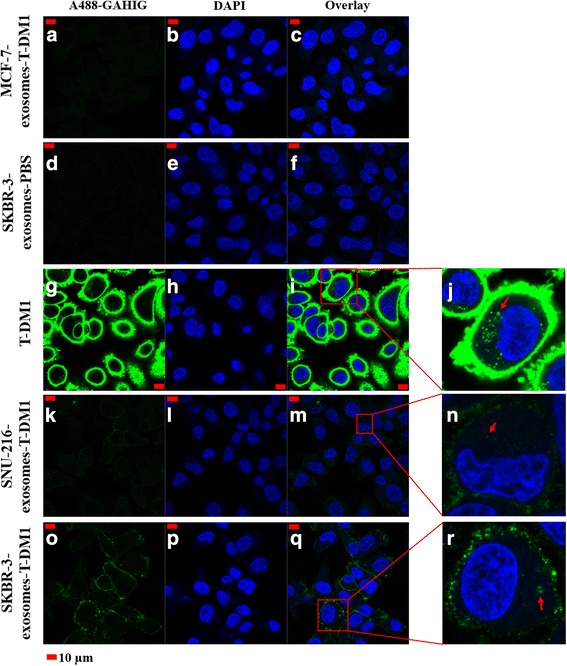
T-DM1 carried on Type A exosomes may bind to cells and internalize. Confocal microscopy images of HER2-positive SKBR-3 breast cancer cells treated with Type A exosomes or unbound T-DM1. T-DM1 was stained with Alexa 488-goat anti-human-IgG (A488-GAHIG) and the nuclei with DAPI. No T-DM1 is detected in SKBR-3 cells when treated with MCF-7 exosomes treated with T-DM1 (**a**–**c**) or when treated with SKBR-3 exosomes treated with PBS (**d**–**f**) (negative controls). Treatment of SKBR-3 cells with unbound T-DM1 (positive control) resulted in strong cell membrane staining (**g**–**i**) and internalization of T-DM1 (insert **j**). Treatment of SKBR-3 cells with SNU-216 exosomes treated with T-DM1 (**k**–**m**) or with SKBR-3 exosomes treated with T-DM1 (**o**–**q**) resulted in cell membrane staining, and internalization of some T-DM1 (inserts **n** and **r**, respectively). The images were taken with identical microscope settings

We next treated HER2-positive SKBR-3 breast cancer cells with Type A exosomes to find out whether exosome-carried T-DM1 may be taken up by the cells. T-DM1 was used as a positive control, and MCF-7 exosomes treated with T-DM1, FBS exosomes treated with T-DM1, SKBR-3 exosomes treated with PBS, and SNU-216 exosomes treated with PBS were used as negative controls. After treating the cells with either Type A exosomes or T-DM1, T-DM1 was stained with Alexa 488-goat anti-human-IgG and the cell nuclei with DAPI, and the slides were viewed using confocal microscopy. Cell membrane binding or internalization of T-DM1 could not be detected in any of the negative control samples (Fig. 3a to f; Additional file 2: Figure S2 panels A to F), whereas strong cell membrane binding of T-DM1 and intracellular T-DM1 were visible in SKBR-3 cells treated with unbound T-DM1 used as the positive control (Fig. 3g to j). Both cell membrane and internalized T-DM1 were also present in SKBR-3 cells that were treated with SNU-216 exosomes treated with T-DM1, or with SKBR-3 exosomes treated with T-DM1, although the amounts of T-DM1 were lower than in the positive control samples (Fig. 3k to n and o to r).

### Type A T-DM1-exosomes reduce viability of HER2-positive cancer cells

**Fig. 4:**
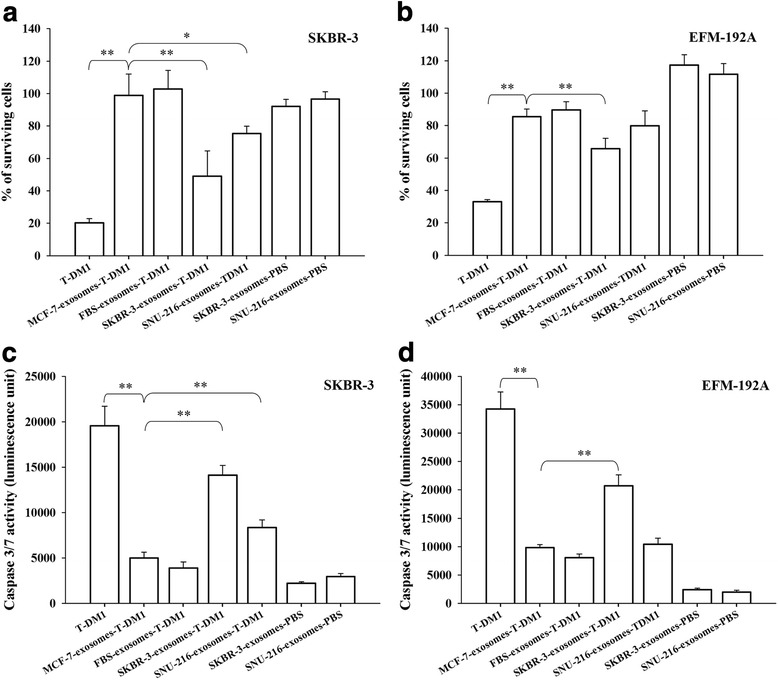
The influence of Type A T-DM1 exosomes on breast cancer cell viability and caspase activity. The effects of Type A exosomes derived from MCF-7, SKBR-3, and SNU-216 cell lines, and from FBS, on the viability of SKBR-3 cells (**a**, **c**) and EFM-192A cells (**b**, **d**) were investigated. Efficacy of other treatments were compared to MCF exosomes treated with T-DM1. Treatment with unbound T-DM1 was used as a positive control. **a**, **b** Treatment with SKBR-3 exosomes treated with T-DM1 reduced the proportion of surviving cells significantly in both cell lines, and SNU-216 exosomes treated with T-DM1 reduced the viability of the SKBR-3 cells. **c**, **d** T-DM1 (positive control) and SKBR-3 exosomes treated with T-DM1 increased the caspase-3 and/or caspase-7 activity in both SKBR-3 and EFM-192A cells compared to MCF-7 exosomes treated with T-DM1. **c** SNU-216 exosomes treated with T-DM1 increased the caspase activity in the SKBR-3 cell line. **, *P* < 0.01; *, *P* < 0.05

The effects of Type A exosomes on cell viability were studied in two HER2-positive breast cancer cell lines that are sensitive to T-DM1, SKBR-3, and EFM-192A [6]. MCF-7 exosomes treated with T-DM1 were selected as a reference for efficacy, since some T-DM1 was detected in this sample by Western blotting and flow cytometry. Treatment with unbound T-DM1 was used as the positive control. After 96 h of incubation, unbound T-DM1 reduced markedly the proportion of surviving cells in both SKBR-3 and EFM-192A cell lines as compared with MCF-7 exosomes treated with T-DM1 (*P* < 0.01). SKBR-3 exosomes and SNU-216 exosomes treated with T-DM1 reduced significantly the viability of SKBR-3 cells (*P* < 0.01, and *P* < 0.05, respectively). SKBR-3 exosomes treated with T-DM1 reduced significantly also the viability of EFM-192A cells (*P* < 0.01), while SNU-216 exosomes treated with T-DM1 did not (Fig. 4a–b).

### T-DM1-treated Type A exosomes increase caspase activity in HER2-positive breast cancer cells

The influence of Type A exosomes on caspase-3 and/or caspase-7 activation was studied in the HER2-positive cell lines SKBR-3 and EFM-192A after 96 h incubation of the cells with Type A exosomes. Efficacy of other treatments were compared to MCF-7 exosomes treated with T-DM1. T-DM1 treatment (positive control) resulted in a marked activation of caspase-3 and/or caspase-7 activity in both cell lines as compared with MCF-7 exosomes treated with T-DM1 (*P* < 0.01). SKBR-3 exosomes treated with T-DM1 increased significantly caspase-3 and/or caspase-7 activity in both SKBR-3 and EFM-192A cells (*P* < 0.01). Exosomes derived from SNU-216 cells and treated with T-DM1 increased significantly caspase activity in the SKBR-3 cells (*P* < 0.01), but not in EFM-192A cells. No change in the caspase activity was detected when the cells were treated with FBS exosomes treated with T-DM1 (Fig. 4c–d).

### T-DM1 is present on newly formed exosomes harvested from the cell culture medium of breast cancer cells previously treated with T-DM1 (Type B exosomes)

We next investigated the properties of exosomes that were not directly mixed with T-DM1, but were newly formed and originated from cells that had been exposed to T-DM1 (Type B exosomes). In these experiments, SKBR-3 breast cancer cells were first incubated with either PBS or T-DM1 for 30 min, followed by washing and incubation in an exosome-depleted cell culture medium overnight. The exosomes shed into a fresh exosome-depleted culture medium were then isolated and studied.

**Fig. 5:**
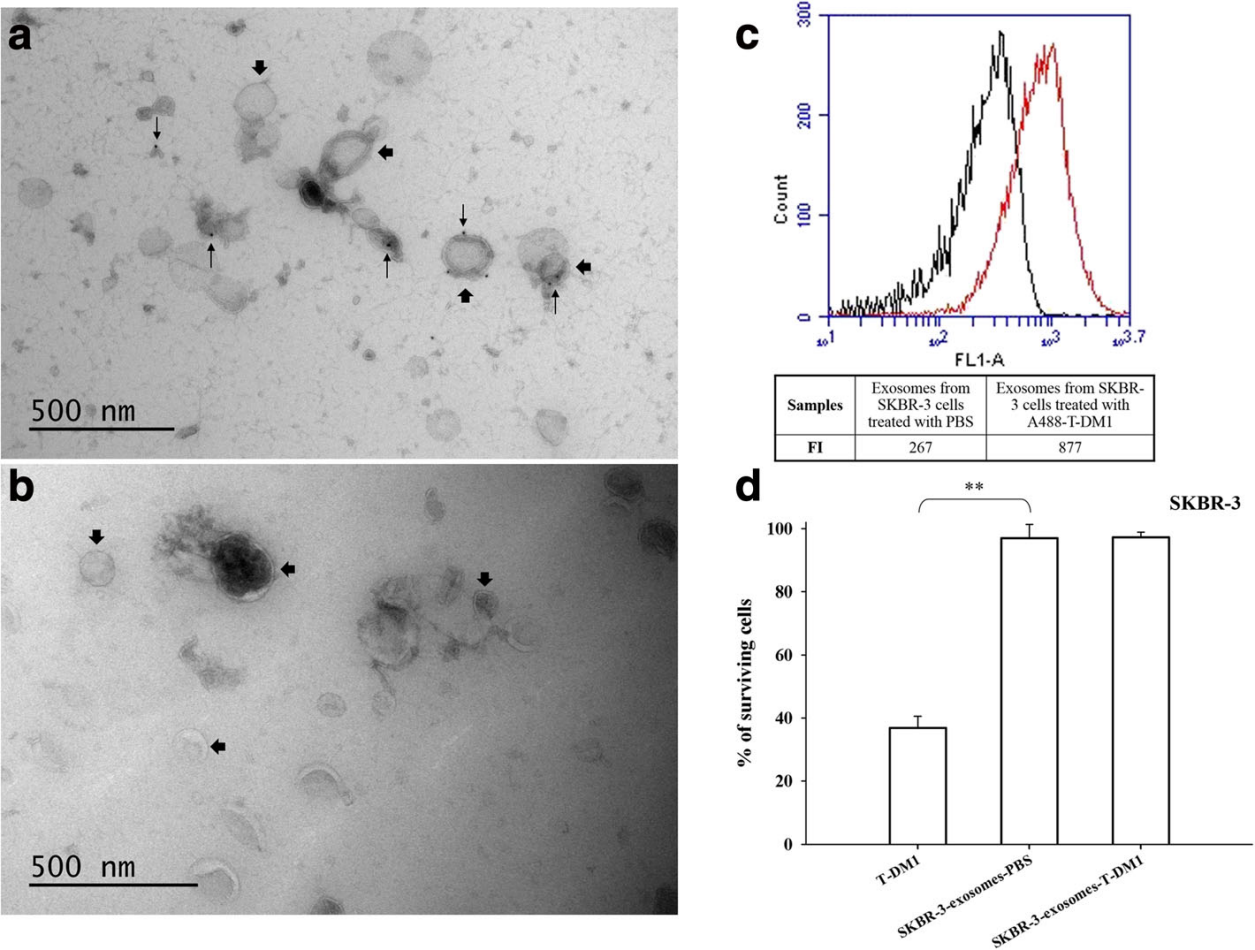
T-DM1 is excreted from SKBR-3 cells on Type B exosomes. **a**, **b** Immuno-electron microscopy images of the exosomes obtained from the cell culture medium of SKBR-3 cells treated with either PBS or T-DM1. Immuno-gold labelled T-DM1 (small arrows) is detectable on exosomes isolated from cells exposed to T-DM1 (**a**), but not when the cells were treated with PBS (**b**). Large arrows point at exosomes, small arrows at T-DM1. **c** In flow cytometry, a higher fluorescent intensity (FI) was detected in exosome samples isolated from the cell culture medium of SKBR-3 cells treated with A488-T-DM1 (red) as compared to samples isolated from the medium of cells treated with PBS (black). **d** T-DM1 reduced markedly the growth of SKBR-3 breast cancer cells (positive control), whereas Type B exosomes secreted into the exosome-depleted growth medium from T-DM1-treated or PBS-treated SKBR-3 cells did not. **, *P* < 0.01

In transmission electron microscopy, exosomes with 30 to 300 nm in size were present in the fresh culture media. Immuno-gold labelled T-DM1 was found on Type B exosomes harvested from the medium of SKBR-3 cells that had been treated with T-DM1, but not when they had been treated with PBS, showing that SKBR-3 cells dispose T-DM1 on exosomes (Fig. 5a and b). When the cell culture medium exosome content was measured with flow cytometry after the cells had been exposed to either A488-T-DM1 or PBS for 30 min, a higher fluorescence intensity was present in the samples obtained from the medium of T-DM1-treated cells as compared to PBS-treated cells (Fig. 5c).

**Fig. 6:**
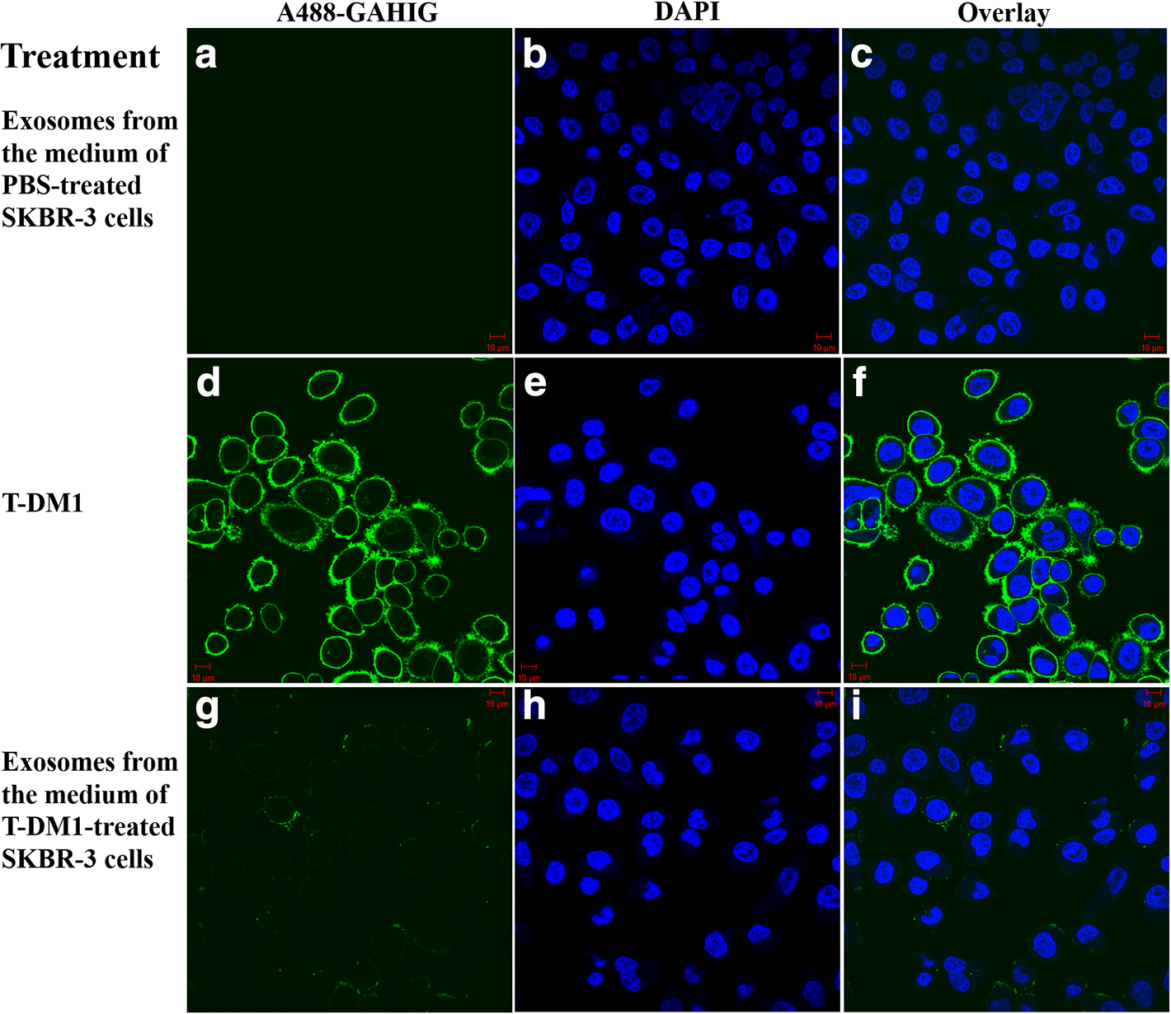
Confocal microscopy images of HER2-positive SKBR-3 breast cancer cells treated with Type B exosomes. **a**–**c** No T-DM1 is detected on the SKBR-3 cells treated with exosomes harvested from fresh cell culture medium of PBS-treated SKBR-3 cells (negative control). **d**–**f** Intense membranous staining is present when the cells were treated with T-DM1 (positive control). **g**–**i** T-DM1 is present on cells treated with exosomes obtained from fresh cell culture medium of T-DM1-treated cells. The images were taken with identical microscope settings

In confocal microscopy imaging, SKBR-3 breast cancer cells showed no staining when treated with exosomes isolated from a fresh medium of SKBR-3 cells previously treated with PBS, whereas the cells stained for T-DM1 when they were treated with exosomes obtained from a fresh medium of SKBR-3 cells that had been previously exposed to T-DM1 (Fig. 6). Exosomes harvested from the cell culture medium of SKBR-3 cells that had been treated with PBS or T-DM1 did not have detectable influence on the viability of SKBR-3 cells (Fig. 5d).

## Discussion

Trastuzumab emtansine (T-DM1) has several proposed mechanisms of action [4]. Active catabolites of DM1 are potent microtubule inhibitors that cause impaired intracellular trafficking and/or cell cycle arrest, which lead to apoptotic cell death or a mitotic catastrophe [1, 6]. T-DM1 also evokes antibody-dependent cell-mediated cytotoxicity (ADCC) and inhibits shedding of the extracellular domain of HER2 [6, 23]. The target of T-DM1, HER2, may be present on cancer-derived exosomes [15, 16], and, in line with the current findings, trastuzumab may bind to HER2-positive exosomes [24]. We hypothesized that T-DM1 may bind to HER2-expressing cancer-derived exosomes. Since exosomes can transfer their contents to both the neighboring cells and distant cells [10, 12], T-DM1-exosomes might be able to carry the cytotoxic agent (DM1) into other cells, which might influence the efficacy and toxicity of T-DM1.

To test this hypothesis, we applied two different strategies for exosome isolation and treatment of cancer cells with exosomes, each of which could have biological relevance in the tumor microenvironment. First, exosomes were harvested from the cell culture medium of HER2-positive breast and gastric cancer cells. The exosomes were then treated with T-DM1 followed by removal of the unbound T-DM1 (Type A exosomes). In this model, T-DM1 binds to cancer-derived exosomes that have already been secreted into the tumor microenvironment. We found T-DM1 on exosomes derived from HER2-positive breast and gastric cancer cells and treated with T-DM1, and showed that these exosomes can carry T-DM1 into other cancer cells leading to caspase activation and cell death. In the second model, cancer cells were treated with T-DM1, washed, and grown in an exosome-free culture medium overnight, and we then isolated the freshly secreted exosomes from the culture medium (Type B exosomes). In this model, T-DM1 first needs to bind to HER2 on cancer cell plasma membrane, be internalized, and then be secreted from the cells into the cell environment on cancer cell-derived exosomes. T-DM1 was found to be present also on Type B exosomes, and, moreover, these exosomes could also carry T-DM1 to other cancer cells.

At the first glance, it might seem unnecessary to make a distinction between Type A and Type B exosomes. The mechanisms that lead to T-DM1 being a part of the Type B exosomes are incompletely understood. Exosomes are secreted when the intracellular multivesicular bodies fuse with the plasma membrane, but the exosomes have a lipid bilayer which has a different composition from the plasma membrane [25]. Therefore, T-DM1 coupled to the plasma membrane may not bind to exosomes during exosome shedding, but T-DM1 more likely needs first to bind to cell surface HER2, be internalized and transported into the early lysosomes, and later into the multivesicular bodies where it becomes integrated into the exosomes and becomes shedded. Alternatively, T-DM1 bound to HER2 on the cell surface could be secreted on microvesicles directly from the plasma membrane. These could be mechanisms with which the cells can dispose T-DM1. If unbound T-DM1 is still present in the cell microenvironment at the time of exosome shedding, it may still bind to Type B exosomes. Little is still known about whether the T-DM1 intracellular cycling changes the biological properties of the exosomes produced after exposure to T-DM1.

The cytotoxic effect of T-DM1 in cells likely depends on the intracellular concentration of DM1, resulting from the breakdown of the antibody part of T-DM1 and the release of DM1 from the lysosomes [4], whereas exosomes lack intraexosomal compartments and do not have acidic pH [26], suggesting that the effect of T-DM1-exosomes on cancer cells results from T-DM1 bound onto the exosome surface rather that from the release of DM1 within the exosome. Exosomes with more HER2 on their surface may have a greater cell growth inhibitory effect than exosomes with less HER2 expression, since they can carry more T-DM1 into the recipient cells. SKBR-3 cells express more HER2 than SNU-216 cells [17], and hence SKBR-3-derived exosomes may also express more HER2 than SNU-216-derived exosomes. We found SKBR-3-derived exosomes to carry more T-DM1 than exosomes derived from the SNU-216 cells. This may explain the observation that Type A T-DM1-exosomes derived from SKBR-3 cells had a greater growth inhibitory effect on both SKBR-3 and EFM-192A breast cancer cells than Type A T-DM1-exosomes derived from SNU-216 cells. Similarly, the treatment with Type A T-DM1-exosomes derived from SKBR-3 cells resulted in a more substantial caspase activation in both SKBR-3 and EFM-192A breast cancer cells than Type A T-DM1-exosomes derived from SNU-216 cells. Since we incubated Type A exosomes with a relatively high concentration of T-DM1 (25 μg/mL), the T-DM1 binding sites of the existing exosomes likely became saturated, which might not have been the case with the newly formed (Type B) exosomes, which could in part explain why Type B exosomes did not have a detectable effect on cancer cell growth unlike the Type A exosomes.

Exosomes may carry mRNA, miRNA, DNA, and proteins to distant cells, which is a type of cell-cell communication [10–12]. We found that exosomes secreted from cancer cells may pick up molecules (such as T-DM1) in the extracellular environment and deliver them into other cells, and the molecules picked up may influence the function of the recipient cells (such as T-DM1 caused growth inhibition and increased caspase activation). This finding suggests that exosomes may regulate cell-cell communication not only by carrying biologically active molecules originating from the intracellular space, but also by carrying molecules originating from the extracellular microenvironment.

The mechanisms of cell uptake of exosomes and their cargo are not well understood. Uptake can happen via the fusion of exosome and the recipient cell plasma membranes, through endocytosis, macropinocytosis, and phagocytosis [27, 28]. Cancer-derived exosomes can enter the circulation and deliver their contents to distant cells [10, 12]. Therefore, T-DM1-exosomes might be able to carry DM1 into distant cells, which could potentially influence even the adverse effects of T-DM1. This hypothesis warrants further study.

## Conclusion

In conclusion, we showed that T-DM1 can bind to exosomes derived from HER2-positive breast cancer cells and gastric cancer cells. In addition, T-DM1 can be delivered on exosomes to other cancer cells, which may result in growth inhibition and apoptotic death of the recipient HER2-positive cancer cells. The results suggest a new, exosome-mediated mechanism of action for T-DM1 that might influence the efficacy of T-DM1. The present observations warrant further study both in in vivo models and in the clinical setting.

## Additional files

Additional file 1: **Figure S1.** Immuno-electron microscopy images showing (A) FBS exosomes treated with T-DM1, and (B) SNU-216 exosomes treated with PBS (Type A exosomes). No T-DM1 is detectable on the exosomes. (TIF 1108 kb)

Additional file 2: **Figure S2.** Confocal microscopy images of SKBR-3 cells. (A-C) No T-DM1 is detectable on cells exposed to FBS exosomes treated with T-DM1, or (D-F) exposed to SNU-216 exosomes treated with PBS (Type A exosomes). (TIF 432 kb)

## Abbreviations

A488: Alexa Fluor 488
ADCC: Antibody-dependent cellular cytotoxicity
BSA: Bovine serum albumin
DM1: Derivative of maytansine
FBS: Fetal bovine serum
FDA: Food and drug administration
FI: Fluorescence intensity
GAHIG: Goat anti-human immunoglobulin
HER2: Human epidermal growth factor receptor-2
PBS: Phosphate-buffered saline
RT: Room temperature
SMCC: *N*-succinimidyl-4-(N-maleimidomethyl) cyclohexane-1-carboxylate
T-DM1: Trastuzumab-emtansine
