# Supplementary figures and images for: Cancer-derived exosomes from HER2-positive cancer cells carry trastuzumab-emtansine into cancer cells leading to growth inhibition and caspase activation
Source: BMC Cancer. 2018 May 2;18:504. doi: 10.1186/s12885-018-4418-2 (PMC5930687; doi:10.1186/s12885-018-4418-2)

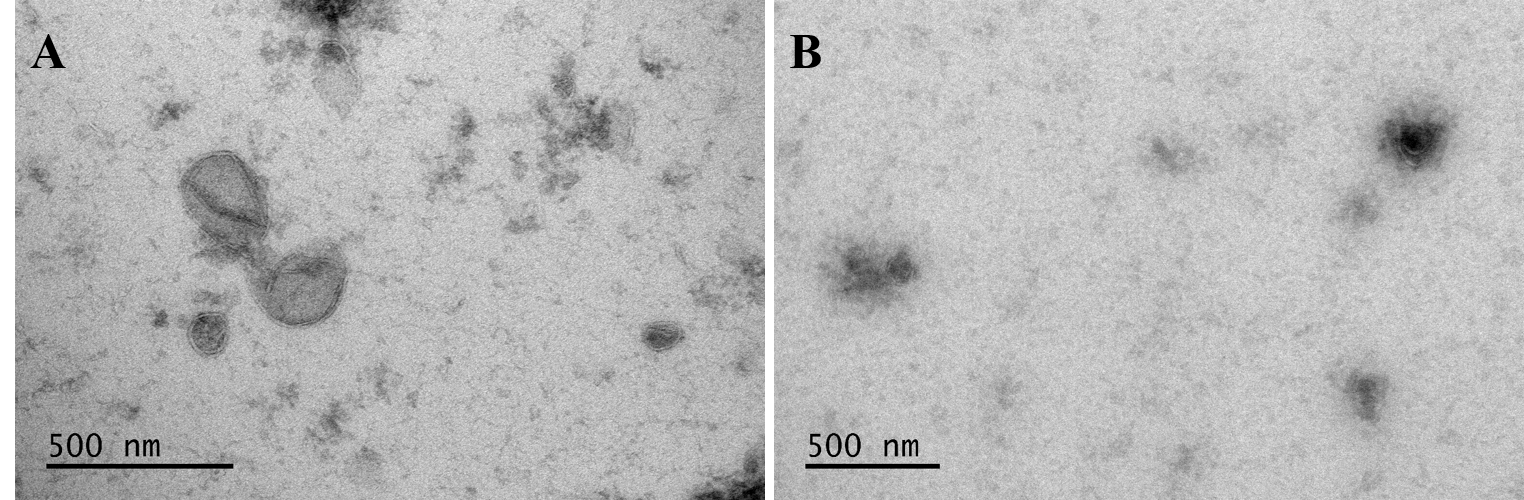

Supplement: Supplementary file 1 — Figure S1. Immuno-electron microscopy images showing (A) FBS exosomes treated with T-DM1, and (B) SNU-216 exosomes treated with PBS (Type A exosomes). No T-DM1 is detectable on the exosomes. (TIF 1108 kb) [file 12885_2018_4418_MOESM1_ESM.tif]

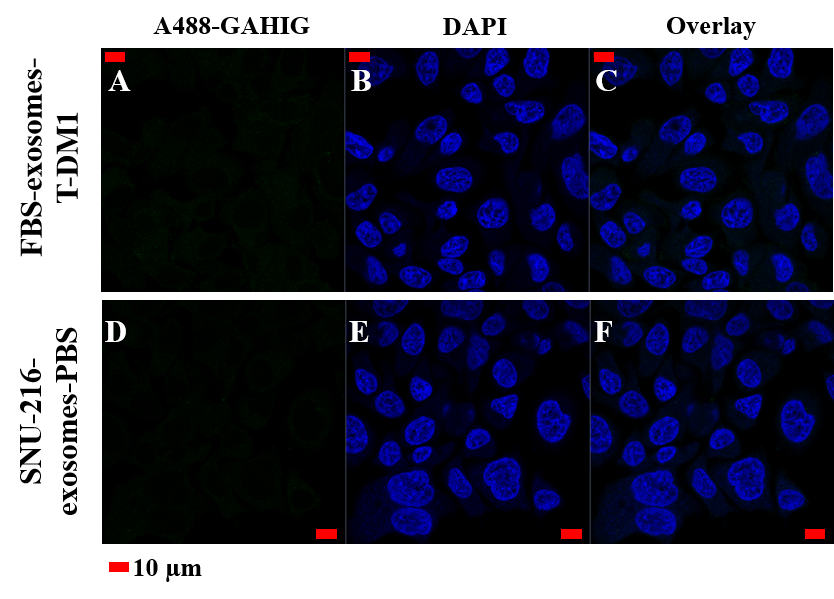

Supplement: Supplementary file 2 — Figure S2. Confocal microscopy images of SKBR-3 cells. (A-C) No T-DM1 is detectable on cells exposed to FBS exosomes treated with T-DM1, or (D-F) exposed to SNU-216 exosomes treated with PBS (Type A exosomes). (TIF 432 kb) [file 12885_2018_4418_MOESM2_ESM.tif]
